# Post-interval EEG activity is related to task-goals in temporal discrimination

**DOI:** 10.1371/journal.pone.0257378

**Published:** 2021-09-27

**Authors:** Fernanda Dantas Bueno, André Mascioli Cravo

**Affiliations:** Center for Mathematics, Computing and Cognition Universidade Federal do ABC (UFABC), São Bernardo do Campo, SP, Brazil; University of Glasgow, UNITED KINGDOM

## Abstract

Studies investigating the neural mechanisms of time perception often measure brain activity while participants perform a temporal task. However, several of these studies are based exclusively on tasks in which time is relevant, making it hard to dissociate activity related to decisions about time from other task-related patterns. In the present study, human participants performed a temporal or color discrimination task of visual stimuli. Participants were informed which magnitude they would have to judge before or after presenting the two stimuli (S1 and S2) in different blocks. Our behavioral results showed, as expected, that performance was better when participants knew beforehand which magnitude they would judge. Electrophysiological data (EEG) was analysed using Linear Discriminant Contrasts (LDC) and a Representational Similarity Analysis (RSA) approach to investigate whether and when information about time and color was encoded. During the presentation of S1, we did not find consistent differences in EEG activity as a function of the task. On the other hand, during S2, we found that temporal and color information was encoded in a task-relevant manner. Taken together, our results suggest that task goals strongly modulate decision-related information in EEG activity.

## Introduction

Perceptual timing is essential for humans and other animals to interact with their environments. A commonly used task to study this ability is temporal discrimination, in which participants have to judge whether a given duration is shorter or longer than a reference. Several studies have compared temporal discrimination tasks with discrimination of other attributes, such as color [1,2], size [3], space [4] and numerosity [5]. For example, Coull and colleagues [1], using functional Magnetic Resonance Imaging, found a higher activation of areas such as the pre-SMA and a network of other cortical and striatal areas when participants paid more attention to the duration than the color of a stimulus. In another study, Kulashekhar and colleagues [2] used a similar design combined with MEG to investigate possible neural correlates in temporal processing. Studies involving color discrimination as a contrast task change color dynamically to make the tasks more cognitively comparable. The rationale is that in both conditions, participants need to keep track of the visual stimulus presented. However, by changing color dynamically, time becomes relevant to the task: now, participants need to track for how long the target was presented with each hue. This limitation makes it hard to dissociate what aspects of neural activity are associated with temporal discrimination or with general task-related decisions in which time is relevant.

Here, we aimed to examine human electroencephalogram (EEG) using a duration and color discrimination task. We used a static display in the color task so participants wouldn’t use the temporal information to estimate the color. As in the study of Coull and colleagues [1], we controlled how much attention was allocated to different dimensions (time or color) by informing participants whether they would make a judgment about time or color before the stimuli (Pure Blocks) or only after the end of the trial (Mixed Blocks). Contrary to previous experiments, we parametrically varied the difference in time and color between stimuli, allowing an in-depth investigation of whether information about time or color in EEG activity was task-dependent. Thus, we aim to compare a simple interval discrimination task to a non-temporal task, in which time is not relevant to the decision.

Different time-resolved M/EEG markers have been proposed to be correlated with temporal judgments, such as the classical contingent negative variation (CNV) [6], the early post-interval N1P2 component [6], and the late positive component (LPC) [7–9]. This last marker has been studied in different explicit temporal paradigms, such as temporal bisection [7,8,10], temporal generalization [7,11], and temporal discrimination [3,9,12–14]. However, different studies have used the LPC to refer to EEG activities diverse in time, topography, and task-related modulations. While some authors identified the LPC at prefrontal electrodes [3,9,11], others have placed it at centro-parietal electrodes [7,10]. Here, we used a combination of multivariate methods of Linear Discriminant Contrast (LDC) [15] and Representational Similarity Analysis (RSA) [16] to evaluate dissimilarities of different activations of the time-resolved EEG signal in the different contexts of the experiment. With this method, it is not necessary to make a priori choices about electrodes and time points to analyse. Since the LDC is a dissimilarity measure across sensors, small changes in different electrodes will contribute to the final estimation, while choosing a set of electrodes for a simple event-related potential analysis might lose critical information.

In summary, we compared distances between patterns of EEG activity and investigated how these distances were modulated by task, by duration, or by color and whether possible modulations depended upon the task to be performed. We found weak differences between tasks when participants were exposed to the duration or color to be stored for further comparison. However, during the presentation of the comparison event, there were clear task-dependant differences in EEG activity. Decisions about durations and color evoked different patterns of EEG activity, at different moments and were modulated more strongly by task-relevant information.

## Materials and methods

### Participants

Twenty-one human volunteers (age range, 21–32 years; 11 women) were informed about the experiment and gave written consent to participate in the experiment. All of them had normal or corrected-to-normal vision and did not report any psychological or neurological diagnoses. The Research Ethics Committee of the Federal University of ABC approved the experimental protocol (CAEE: 38370314.0.0000.5594), and the experiment was performed following the approved guidelines and regulations. Data from one volunteer (age 24, female, not included in the twenty-one participants above) were excluded from the analyses due to excessive noise and artifacts in the EEG signal (proportion of rejected trials above 20% in two segment windows of analyses as explained below).

### Stimuli and procedures

**Fig 1:**
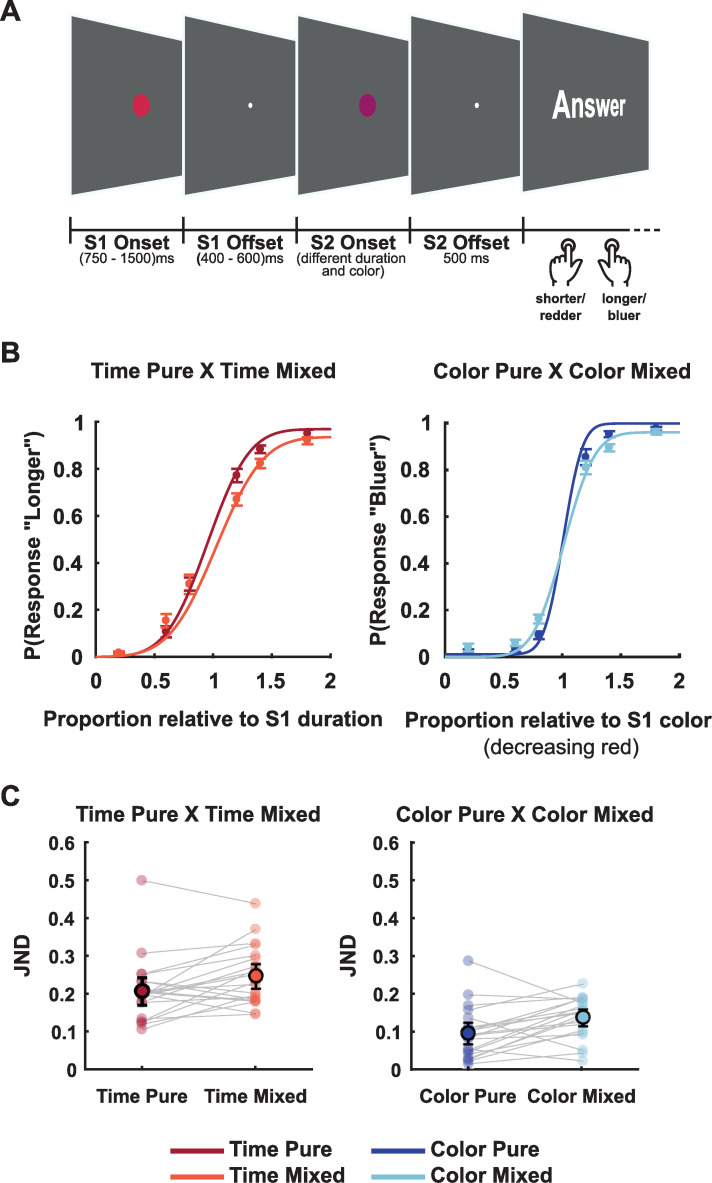
Experimental design and behavioral results. (A) Temporal/color discrimination task. The figure represents the time course of one trial. Before each block, a written cue would indicate if it was a ‘Time’ for Time Pure blocks, ‘Color’ for Color Pure blocks, or ‘Time/Color’ for Mixed blocks. (B) Curves show the psychometric functions for each condition, depicting the proportion of responding ‘longer’ for Time Pure and Time Mixed conditions (left) and responding ‘bluer’ for Color Pure and Color Mixed conditions (right). (C) Just Noticeable Difference (JND) by condition. Faint-colored filled circles represent individuals’ JND by condition. Lines connect JNDs for different conditions for each individual. Sharp-colored filled circles represent the mean JNDs, and bars represent the standard error of the mean.

The experiment consisted of a duration or color discrimination task (Fig 1). The stimuli were presented using Psychtoolbox [17] v.3.0 package for MATLAB on a 17-inch CRT monitor with a vertical refresh rate of 60 Hz, placed approximately at a viewing distance of 50 cm from the participant. Responses were collected via a response box of 9 buttons (DirectIN High-Speed Button; Empirisoft). We used the left and right buttons for responses in which participants should respond using both hands. We presented 720 trials consisting of two visual stimuli (filled circles) with different colors and durations. Participants were instructed to answer if the second stimulus was shorter/longer in duration or redder/bluer than the first one. The magnitude to judge was determined by the block condition: (1) In Time Pure and Color Pure blocks (2 blocks of each), participants were informed beforehand whether to judge differences in duration or color between the two visual stimuli; (2) In Mixed blocks (the remaining four blocks), participants would only know the magnitude (time or color) to judge during the response screen, 500ms after the offset of the second stimulus. Block order was randomized for all participants, and the background color was gray (RGB-color 100; 100; 100).

Each trial started with the presentation of a circle (S1, one visual degree radius) at the center of the screen with a duration randomly chosen between 750 ms to 1500 ms, and colored in the RGB space [1-C, 0, C], in which C could range randomly from 0.2 to 0.5. The RGB space and parameter C for manipulating color was chosen based on [18]. After a random ISI of 400 ms to 600 ms, in which only a fixation point was present (0.25 visual degree radius), a second circle (S2, one visual degree radius) appeared with a different duration and color. Duration and color (controlled by parameter C) of S2 could range from 0.2 to 1.8 times the duration and color of S1 within six possibilities in total: 0.2, 0.6, 0.8, 1.2, 1.4, 1.8. Durations and colors were independently randomized, and thus, orthogonal. After a delay of 500 ms, a response screen was presented in which participants were instructed to judge the duration or color of S2 relative to S1. In Pure blocks, the response screen reminded participants which dimension to be compared, while in Mixed blocks, the response screen informed which dimension should be compared.

### EEG recordings and pre-processing

EEG was recorded continuously from 64 ActiCap Electrodes (Brain Products) at 1000 Hz by a QuickAmp amplifier (Brain Products). All sites were referenced to FCz and grounded to AFz. The electrodes were positioned according to the International 10–10 system. Additional bipolar electrodes registered the electrooculogram (EOG). Data pre-processing was carried out using FieldTrip [19] Toolbox for MATLAB. We segmented the data in four different epochs, for the period during the presentation of S1 and S2 (S1/S2 onset analysis) and just after the offset of each stimulus (S1/S2 offset analysis). The results section will refer to S1 onset and offset as exposure phase, and S2 onset and offset as the decisional phase. Filters were applied to the continuous data with a bandpass of 0.1 Hz to 30 Hz (Butterworth filter, order 3). All data were re-referenced to the activity of electrodes TP9 and TP10, located in the earlobes and downsampled to 256 Hz.

For the S1/S2 onset analysis, epochs were locked at the onset of S1/S2, and data were segmented from -150 ms to 750 ms. For the S1/S2 offset analysis, epochs were locked at the offset of S1/S2, and data were segmented from -150 ms to 400 ms for S1, and to 500 ms for S2. Channels with missing data due to problems in acquisition or channels with excessive noise were interpolated with neighbor channels using the FieldTrip channel repair function. Data from most participants had none or up to two channels interpolated. Only two participants had 3 and 4 channels interpolated.

For eye movement artifact rejection, an independent component analysis (ICA) was performed. Eye-related components were identified with the help of SASICA available for FieldTrip [20] and by visual inspection of topographies and time series from each component. Eye related components were then rejected for all segments. Baseline correction was performed using the periods from 150 ms before S1/S2 onset and 50 ms before and 50 ms after S1/S2 offset. Trials that exceed 200 μV for onset segments or 150 μV for offset segments were rejected. The percentage of rejected trials for S1 onset segment was 1.85% (range between 0% - 11.81%), for S2 onset was 1.18% (0% - 6.11%), for S1 offset was 0.75% (0%- 4.58%) and for S2 offset was 0.83% (0% - 4.58%).

### Behavioral analysis

Behavioral analysis was based on the proportions of each type of response (longer/shorter or redder/bluer) as a function of the duration or proportional color of the second stimulus (S2) relative to the first stimulus (S1). We estimated psychometric functions for each participant in different conditions: Time Pure, Color Pure, and for the mixed blocks, we separated the data in trials in which participants were asked about duration (Time Mixed) and color (Color Mixed). Each of the four experimental conditions comprised 180 trials.

We fitted cumulative normal psychometric functions for each participant and condition, defined by four parameters: threshold, slope, lapse-rate, and guess-rate [21]. We used the FitMultiple function from Palamedes Toolbox [22], split by time and color trials in both Pure and Mixed conditions. Guess rates and lapse rates were unconstrained between conditions and restricted to a maximum of 0.15. Each function’s four parameters were fitted using maximum likelihood estimation as implemented in the toolbox [22]. We estimated the Point of Subjective Equality (PSE) and the JND (Just Noticeable Difference) to evaluate participants’ performance. The JND is the difference from 25% to 75% estimates of the psychometric curve, divided by two. This measurement represents how much different one stimulus has to be relative to another so that participants can notice. In contrast, the PSE represents the magnitude difference by which the second stimulus is equally likely to be judged as longer/shorter or redder/bluer than that of a first stimulus. We compared the JND and PSE from the Pure Blocks to their counterparts in the Mixed Blocks using a paired t-test. Effect sizes were estimated using Cohen’s d as implemented in JASP [23], and Bayes Factor (BF_10_) is also reported. Moreover, we calculated the accuracy (hit rate) for each condition and compared it by a two-way ANOVA.

Finally, to explore perceptual interference effects, whether participants used the irrelevant magnitude to estimate the relevant feature, we also performed a binomial regression using the response as the dependent variable and both the time proportional and color proportional as regressors. We evaluate each coefficient separately for each experimental condition.

### Multivariate pattern analysis

To compare the pattern of EEG activity across different conditions, we used Linear Discriminant Contrasts (LDC) [15]. We used this method to estimate distances in the time-resolved EEG signals from different tasks and conditions for each participant. All EEG electrodes were used in this analysis, except for the reference TP9 and TP10). The LDC is a cross-validated Mahalanobis distance and allows the interpretation of ratios between distances, as its null distance is zero [15]. The LDC is calculated as:

$$LDC({EEG}_{j,i},{EEG}_{k,i})={\left({EEG}_{j,i}-{EEG}_{k,i}\right)}_{A}\times pS\times {\left({EEG}_{j,i}-{EEG}_{k,i}\right)}_{B}^{T}$$ (1)

where *EEG*_*j*,*i*_ and *EEG*_*k*,*i*_ are row vectors of the means for each channel of the EEG activity in condition **k** and **j**, respectively, for each time point *ith*. *A* and *B* separate data in different subsets, representing different folds; *pS* is the pseudo inverse covariance matrix between *EEG*_*j*,*i*_ residuals and *EEG*_*k*,*i*_ residuals from subset *A*. We used a shrinkage estimator to calculate the pseudo-inverse covariance matrix *pS* [24,25]. Residuals were calculated by subtracting the activity of each trial, time point, and electrode from the mean activity for that electrode at that time point. The distance estimates are then averaged across all possible cross-validation folds.

Before estimating the LDC, data were smoothed within a 39 ms window. We used two-fold cross-validation to compare the electrophysiological activity during different experimental conditions. Folds were based using the blocked experimental design. For example, to compare EEG signals from pure conditions, we used each condition’s first block as one fold and the remaining block as the other fold. The analysis was conducted at each time point (3.9 ms apart after downsampling). To evaluate the estimated distances and correct for multiple comparisons across time, we used a mass-univariate approach. As suggested by Groppe and colleagues [26], we used a permutation test over the tmax statistic, with strong control of the familywise error rate. All tests were one-sided t-tests compared with zero, and p-values were estimated using 10000 permutations. Significance values were based on an alpha level of 5% and we only considered significant windows ranging more than 20 ms.

We used a Representational Similarity Analysis approach to investigate how different aspects of time or color information influenced electrophysiological activity. LDCs were calculated pairwise and used to create representational dissimilarity matrices (RDMs) for different intervals or color information by condition for each time point independently. We built theoretical matrices that represented the distances for time or color information for different comparisons separately. The resulting pairwise distances of the data and theoretical distances’ matrices were then entered into a simple linear regression analysis, separately by condition and segments of the experiment (S1 or S2, onset or offset) for each time point. The data-derived distances were dependent variables, and the theoretical distances matrices the independent variables. The estimated coefficients were compared to zero using a similar mass univariate approach as described above.

## Results

### Behavioral results

The behavioral results (Fig 1) showed that sensitivity, measured by the JND (Just Noticeable Difference), improved when participants knew beforehand which magnitude they would judge (mean ± standard error of the mean, JND_TimePure_ = 0.206 ± 0.018, JND_TimeMixed_ = 0.246 ± 0.017, t(20) = −2.777, p = 0.012, d = 0.606, BF_10_ = 4.452; JND_ColorPure_ = 0.095 ± 0.014; JND_ColorMixed_ = 0.136 ± 0.012, t(20) = −3.039, p = 0.006, d = 0.663, BF_10_ = 7.271). There was no difference in bias, measured by the Point of Subjective Equality between mixed and pure blocks (PSE_TimePure_ = 0.947 ± 0.025, PSE_TimeMixed_ = 0.981 ± 0.032, t(20) = −1.480, p = 0.154, d = 0.323, BF_10_ = 0.585; PSE_ColorPure_ = 1.023 ± 0.021, PSE_ColorMixed_ = 0.999 ± 0.023, t(20) = 0.986, p = 0.336, d = 0.215, BF_10_ = 0.350). We assessed the goodness of fit using Tjur’s Coefficient of Determination [27] (mean D_TimePure_: 0.59, range 0.24 to 0.85; mean D_TimeMixed_: 0.50, range 0.29 to 0.72; mean D_ColorPure_: 0.80, range 0.39 to 0.98; mean D_ColorMixed_: 0.68, range 0.33 to 0.82).

We evaluated accuracy (proportion of correct responses) for each condition (mean accuracy_TimePure_: 0.86 (range: 0.74 to 0.96); accuracy_TimeMixed_: 0.82 (range: 0.73 to 0.91); accuracy_ColorPure_: 0.94 (range 0.81 to 0.99); accuracy_ColorMixed_: 0.90 (range: 0.77 to 0.95). A two-way ANOVA revealed a significant effect for type of block (pure or mixed: F(1,20) = 42.195, p < .001, ω^2^ = 0.152) and magnitude (time or color: F(1,20) = 75.734, p < .001, ω^2^ = 0.400), but no interaction (F(1.20) = 0.018, p = 0.895, ω^2^ < 0.001). Our behavioral analyses showed that participants prioritized task-relevant information when they could anticipate the task to be performed, leading to a better performance for pure blocks compared to mixed blocks. Also, the accuracy analysis showed that participants performed better at color trials than in duration trials.

To explore perceptual interference effects, we analysed the coefficients from binomial regression between the answer (shorter/redder or longer/bluer) and time or color proportions (separated regressors). The results showed significant effects only for the relevant information. Proportional time coefficients were significantly different than zero for Time Pure (t(20) = 11.254, p < .0001, d = 2.456, BF10 > 100) and Time Mixed (t(20) = 15.186, p < .0001, d = 3.314, BF10 > 100), but for time irrelevant trials in Color Pure (t(20) = -0.414, p = 0.683, d = 0.090, BF10 = 0.246) or Color Mixed (t(20) = 1.122, p = 0.275, d = 0.245, BF10 = 0.396). Proportional color coefficients were significantly different than zero only for color relevant trials in Color Pure (t(20) = 2.582, p = 0.018, d = 0.563, BF10 = 3.131) or Color Mixed (t(20) = 13.820, p < .0001, d = 3.016, BF10 > 100), but not in Time Pure (t(20) = 0.428, p = 0.674, d = 0.093, BF10 = 0.247) or Time Mixed (t(20) = 0.413, p = 0.684, d = 0.090, BF10 = 0.246). These results show that participants did not rely on irrelevant information to discriminate relevant information.

### Electrophysiological results

#### Exposure phase: No consistent differences in EEG activity by task-goals during or after S1

Task-related activity: In a first analysis, we focused on how activity evoked by S1 was modulated by the task to be executed. We aimed to investigate whether paying attention to the duration or the color of the stimuli leads to different stimulus encoding reflected in the EEG signal. Linear Discriminant Contrasts (LDC) were calculated by comparing: i) pure conditions (Time Pure vs. Color Pure), ii) pure versus mixed conditions (Time Pure vs. Time Mixed and Color Pure vs. Color Mixed); and iii) between mixed conditions. The LDC was estimated from 150 ms before to 750ms after S1 onset (given that the shortest possible duration of S1 was 750 ms, this means that all not rejected trials were used). For the S1 offset, we evaluated the EEG signal from 150 ms before S1 offset up to 400 ms (given that the shortest interval between S1 and S2 was 400 ms).

**Fig 2:**
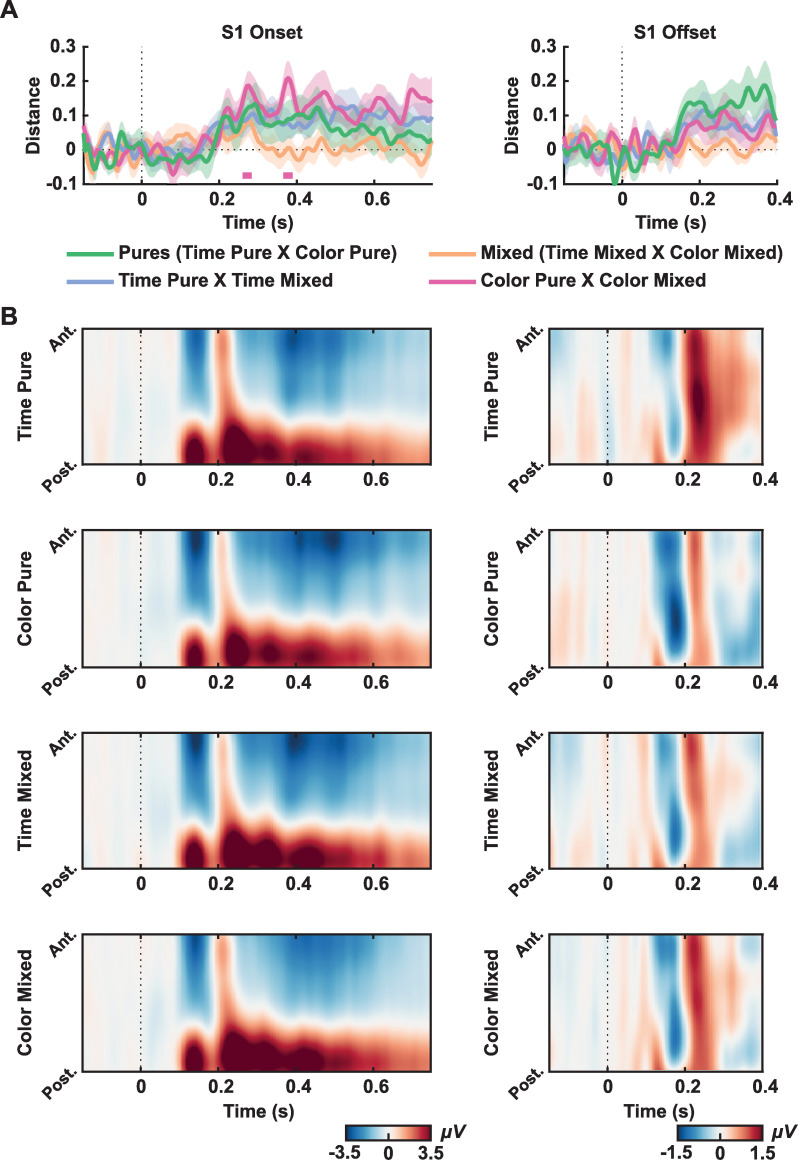
Exposure effects. (A) LDC distances between conditions for S1 onset (left) and S1 offset (right). The green curve indicates the mean distance between pure tasks (distance between Time Pure and Color Pure). The orange curve indicates the mean distance between mixed tasks (Time Mixed and Color Mixed). The purple curve indicates the mean distance between Time Pure condition and Time Mixed. The pink curve indicates the mean distance between Time Pure condition and Time Mixed. Shaded areas indicate the Standard Error of the Mean. Straight lines indicate significant windows for distances from the permutation test. (B) Spatial-temporal ERPs for each condition from S1 onset (right) and S1 offset (left). Graphs show the mean electrical potential between participants from a topographical organization of electrodes (anterior to posterior) in time. All electrodes were used for plotting, except for the reference ones (TP9 and TP10).

The mean distances between tasks during the exposure phase (S1) are shown in Fig 2A. The spatial-temporal distributions of Event-Related Potentials (ERPs) for each task and period are shown in Fig 2B. Although there were short periods in which LDC exceeded the critical t-value (3.4974) in Color Pure vs. Color Mixed conditions, distances between tasks were in general small and not significant (for pure tasks comparison, critical t was 3.4411; Time Pure compared to Time Mixed critical t was 3.5405; for mixed tasks comparison, critical t was 3.4802). We also plotted the ERP for central electrodes for longer than 1300 ms trials for S1 onset to check for the CNV component (S1 Fig, supplementary material). The figures can be found in the supplementary material at OSF (link to OSF).

For S1 offset, there were stronger distances between pure tasks, although none reached statistical significance (for pure tasks comparison, critical t was 3.3870; Time Pure compared to Time Mixed critical t was 3.3990; Color Pure compared to Color Mixed critical t was 3.3647; for mixed tasks comparison, critical t was 3.4991). We show the spatial-temporal distribution of ERPs (Fig 2B) and topographies in S3 Fig.

Time and color-related activity: In a second analysis, we investigated if the duration or color of S1 modulated EEG activity. We aimed to test whether stimuli of different colors or durations evoked different patterns of EEG activity and whether this difference was more robust when that specific dimension was task-relevant. Color information was evaluated in the time-resolved S1 onset signal up to 750 ms. Given that information on how much time has passed since S1 onset was only available at S1 offset, we evaluated time information only at S1 offset. For each participant, S1 duration (from 750 ms to 1500 ms) or its color (indexed by the C parameter) were binned into six bins separately (around 30 trials for each time or color bins, for each experimental condition). The mean duration or mean C for each bin was used to calculate pairwise distances and build theoretical matrices. Pairwise LDC was calculated for each comparison. As explained in the methods section, these resulting pairwise LDC and the theoretical distances’ matrices were then entered into a linear regression analysis. In general, there were no consistent modulations of the EEG signal by color (Time Pure critical t = 3.5422; Color Pure critical t = 3.6045; Time Mixed critical t = 3.5887; Color Mixed critical t = 3.6562). For duration, we found one small period in which coefficients were larger than zero in Time Pure blocks (critical t = 3.5029; from 342.2 ms to 365.6 ms), but not for other conditions (Color Pure critical t = 3.4943; Time Mixed critical t = 3.4868; Color Mixed critical t = 3.4894). As shown in S4 Fig this difference seems to be due to a higher amplitude of a fronto-central activity for shorter durations.

#### Decisional phase: Consistent differences between tasks during and after S2

Task-related activity: In the next step, we focused on the activity evoked by S2, the comparison stimulus. We performed the same LDC analysis to compare tasks during the second stimuli (S2 onset segments). To have a good number of trials of each condition and a considerable amount of time points during S2, this analysis was performed on data from trials in which the second stimulus lasted at least 750 ms.

**Fig 3:**
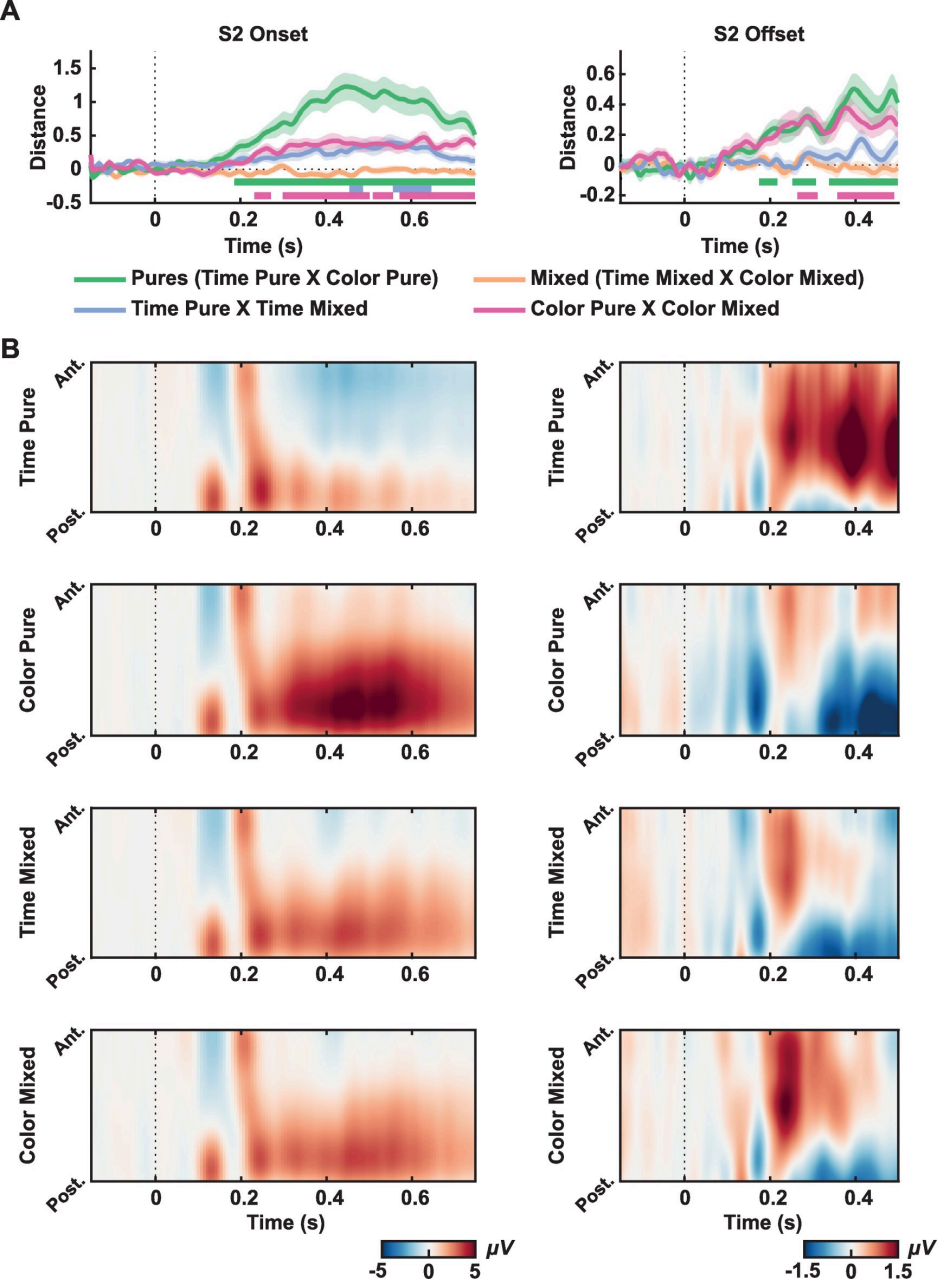
Decisional effects. (A) LDC distances between conditions for S2 onset (left) and S2 offset (right). Different colors indicate different comparisons. Shaded areas indicate the Standard Error of the Mean. Straight lines indicate significant windows for distances from the permutation test. (B) Spatial-temporal ERPs for each condition from S2 onset (right) and S2 offset (left). Graphs show the mean electrical potential between participants from a topographical organization of electrodes (anterior to posterior) in time. All electrodes were used for plotting, except for the reference ones (TP9 and TP10).

For S2 onset, there was a consistent difference in the EEG signal between pure tasks (green line in Fig 3A left, window tested = -150 ms to 750 ms; critical t = 3.3922, significant distances from 185.9 ms to 748.4 ms). We also found differences for Time Pure and Time Mixed (purple line in Fig 3A left, critical t = 3.3722, from 455.5 ms to 486.7 ms, from 557.0 ms to 646.9 ms) and Color Pure to Color Mixed (pink line in Fig 3A left, critical t = 3.3942, from 232.8 ms to 271.9 ms, from 299.2 ms to 502.3 ms, from 510.2 ms to 557.0 ms, and from 572.7 ms to 748.4 ms), but not between mixed conditions, as expected (critical t = 3.5974). The spatial-temporal ERPs illustrate these differences measured by LDC from these conditions (Fig 3B, left). As expected, no significant distance was found between mixed tasks. In all significant comparisons, the difference was strongly driven by a centro-parietal p300 like response present in trials in which participants have to decide on the color of S2 (mixed blocks and pure color blocks). Similar to S1 analysis, we plotted topographies (S5 Fig) and ERPs (S6 Fig) for longer than 1300 ms trials to check for the CNV component. Although not clear in S6 Fig due to the mixture of different intervals, we find a central negative activity for the Time Pure condition, which indicates the development of the CNV component.

The same analysis was conducted for the S2 offset, from 150 ms before the offset of the stimulus to 500 ms after. Trials in which the second stimuli lasted less than 300 ms were excluded from this analysis to reduce sensory ERPs’ contamination. Again, there was a significant distance between pure tasks (green line in Fig 3A right, window tested = -150 ms to 500 ms; critical t = 3.3770, from 174.2 to 217.2 ms, from 252.3 to 307.0 ms and from 338.3 ms to 498.4 ms). There was also a significant distance between Color Pure and Color Mixed (pink line in Fig 3A right, window tested = -150 ms to 500 ms; critical t = 3.3964, from 264.1 ms to 310.9 ms, and from 357.8 ms to 490.6 ms), but not between Time Pure and Time Mixed (critical t = 3.5163) or between mixed tasks (critical t = 3.5266). Differences across conditions were strongly driven by EEG activity present in trials where participants have to decide on the duration of S2 (mixed blocks and pure time blocks). In S7 Fig, we plot the topographies for each condition at S2 offset, and although we can see a positive parietal activity for Time Pure that is different for Color Pure, we also have a strong positive fronto-central activity for the first condition.

#### Time information, but not color, modulate EEG activity for task-relevant conditions at the decisional phase

**Fig 4:**
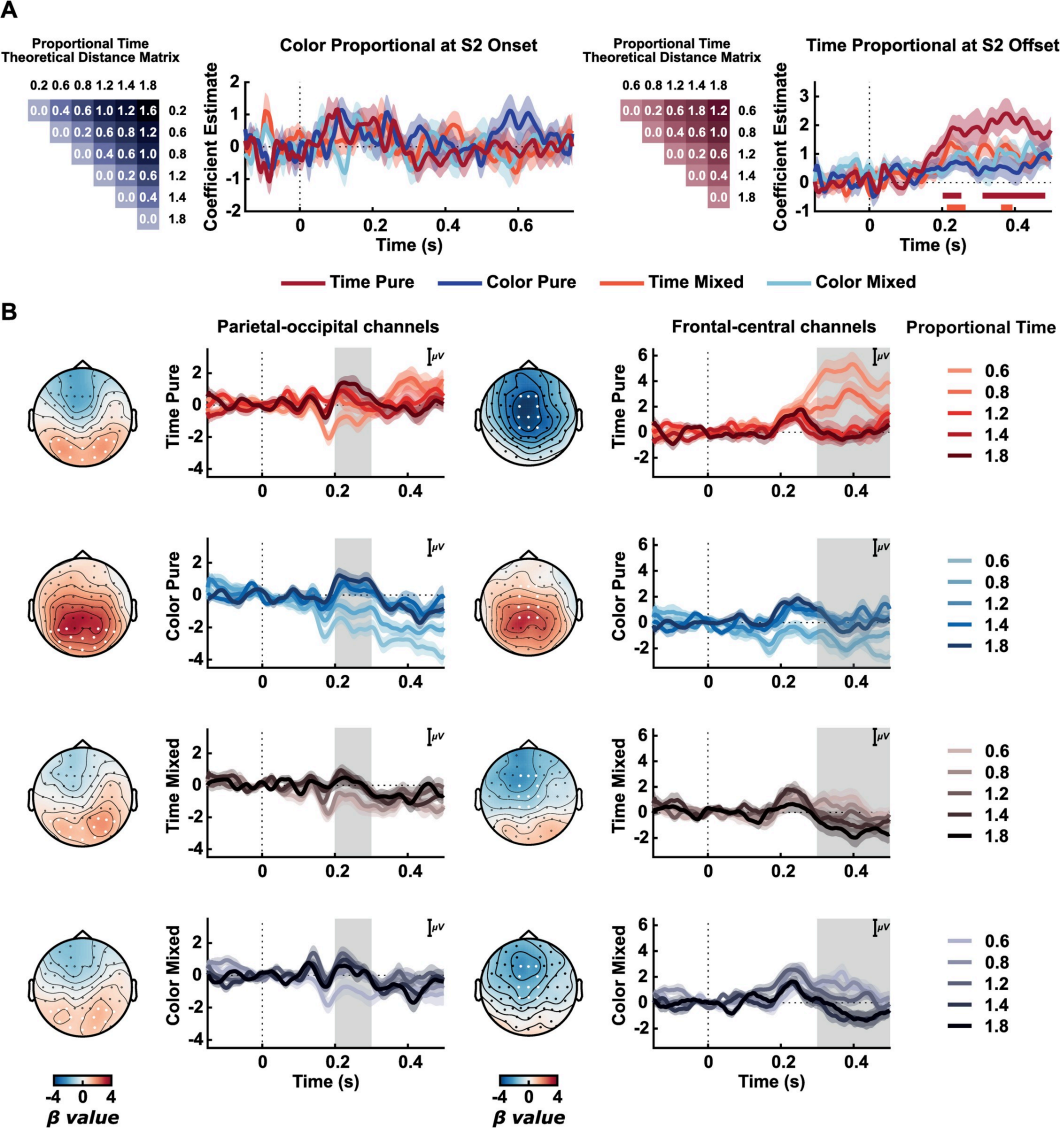
Proportional time and color information at S2 offset. (A) Coefficient estimates from RSA for relative proportional time information at S2 offset (left) and relative proportional color information at S2 onset (right). Matrices depict theoretical models used for RSA for time and color relative from S2 to S1. Curves show mean coefficient values; shaded areas indicate Standard Error of the Mean. Straight Lines below indicate significant windows for RSA’s coefficient estimates for each condition from the permutation test, represented by different colors. (B) Event-Related Potentials for different time proportions and conditions. Topographies of coefficients (β) values from the mass univariate regression analysis are shown for different windows. The first column represents ERPs from the selected parietal-occipital electrodes (marked as white, P8, P6, P4, P3, P5, P7, PO8, PO4, POz, PO3, PO7, O1, Oz, O2) in the 200ms to 300ms window (gray area). The second column represents ERPs from the selected fronto-central electrodes (marked as white, F1, Fz, F2, FC1, FC2, C2, Cz, C1, CP1, CPz, CP2) in the 300ms to 500ms window (gray area).

Decision-related activity: We examined the modulation of EEG activity as a function of the stimulus magnitude of S2 relative to S1. An RSA approach was used to compare activity evoked by stimuli representing different proportions in time (proportional time) or color (proportional color) from S2 to S1, condition-wise. For time information, this analysis was performed on EEG activity of S2 offset (from 150 ms before the offset of the stimulus to 500 ms after) since full temporal information would be available only when the interval had elapsed. For color, this analysis was done for S2 onset up to 750 ms (for trials longer than this duration at S2), and we used the relative proportional color from S2 to S1. We calculated pairwise LDCs of the EEG signal for five possible time proportions of S2 relative to S1 (0.6, 0.8, 1.2, 1.4, 1.8), excluding the 0.2 proportion (see next) at S2 offset. Importantly, the 0.2 time proportion (in duration) was not included, given that this condition had only very short durations (maximum of 300 ms) to avoid false positives due to remaining evoked potentials from the onset and offset of the short duration visual stimulus. We also calculated pairwise LDCs of the EEG signal at S2 onset for all six possible color proportions (0.2, 0.6, 0.8, 1.2, 1.4, 1.8) for trials longer than 750 ms, thus we analysed the results up to this time point. These proportions were used to calculate pairwise distances and build two theoretical matrices, one relative to distances in time and one relative to distances in color (Fig 4A). As before, these matrices were entered into simple linear regression analyses, separately by condition, and coefficient estimates were evaluated.

As can be seen in Fig 4A (left column), we did not find a significant relation between color information and EEG activity at S2 onset (Time Pure critical t = 3.6671; Color Pure critical t = 3.6733; Time Mixed critical t = 3.6349; Color Mixed critical t = 3.6429). Further exploratory ERPs can be found at S8 Fig).

However, for proportional time, we observed increasing coefficient estimates for time-relevant conditions (Fig 4A, right column). The RSA showed an increasing dissimilarity for proportional time information in the Time Pure condition (window tested = -150 ms to 500 ms; critical t = 3.3840, significant time windows from 201.6 to 252.3 ms, and from 310.9 ms to 482.8 ms), and in the Time Mixed (window tested = -150 ms to 500 ms; critical t = 3.4491, from 213.3 ms to 264.1 ms, and from 361.7 ms to 393.0 ms). We did not find statistically significant results for Color Pure (critical t = 3.4498) nor Color Mixed (critical t = 3.3715).

The modulation of EEG activity by proportional time information can be seen in Fig 4B. Based on the RSA analysis and the spatial-temporal evoked activity of S2 offset (Fig 3B, right column), we explored further ERPs at two different windows: from 200 ms to 300 ms and from 300 ms to 500 ms. For each of these periods, a linear regression between time proportions (0.6, 0.8, 1.2, 1.4, and 1.8) and EEG activity was performed for each participant and condition in each electrode and time point. As shown in these topographies (Fig 4B), these two intervals seem to illustrate two stages of post-interval processing. A first parietal-occipital pattern (electrodes: P8, P6, P4, P3, P5, P7, PO8, PO4, POz, PO3, PO7, O1, Oz, O2) that has a positive correlation with time across all conditions. A second fronto-central pattern of activity (electrodes: F1, Fz, F2, FC1, FC2, C2, Cz, C1, CP1, CPz, CP2) shows a negative correlation between time and evoked activity: the shorter the second stimuli is from the comparison, the higher the amplitude of this late stage of this ERP.

**Fig 5:**
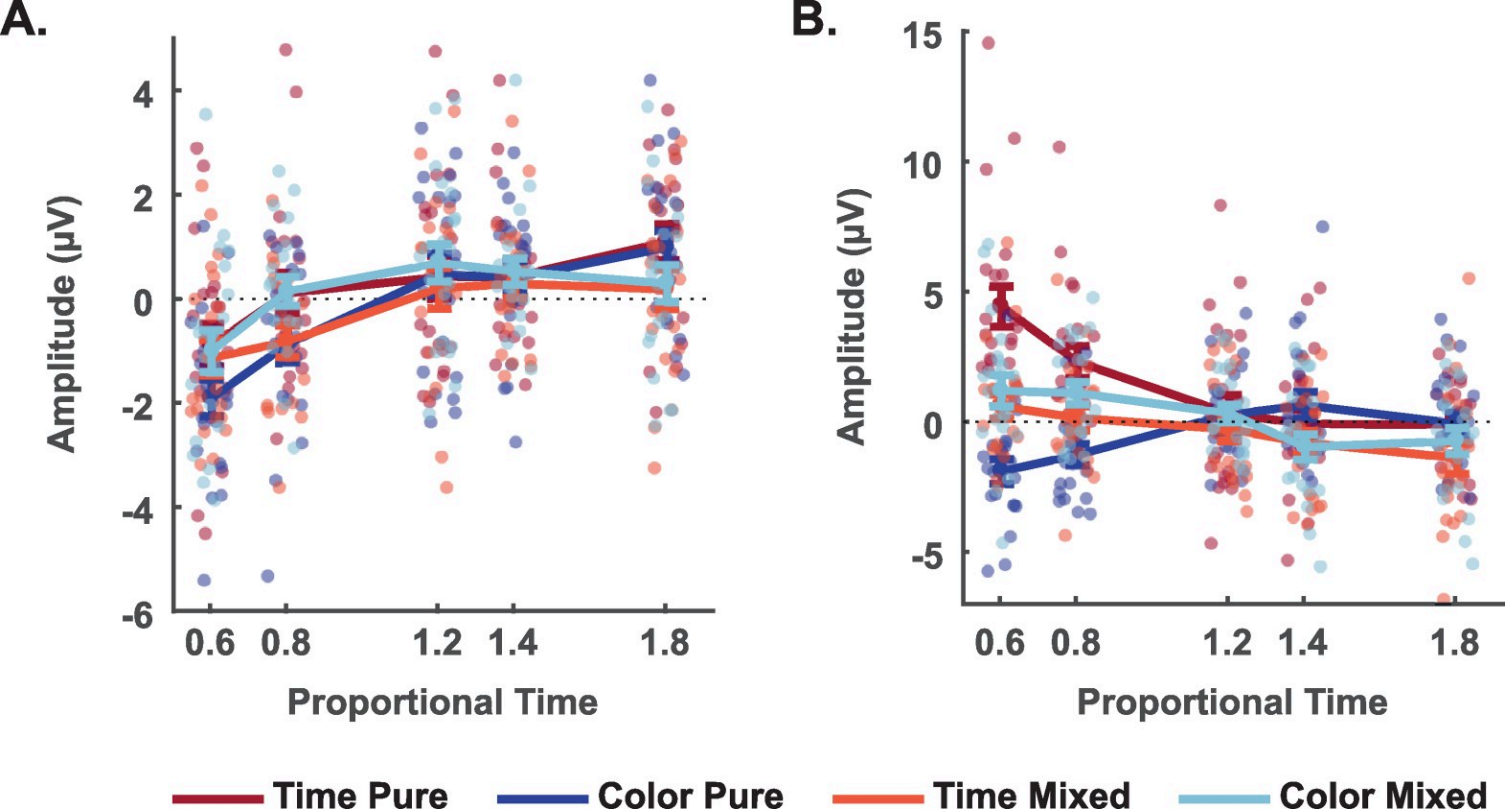
ERP amplitude and proportional time and (A) early post-interval activity at the parietal-occipital electrodes, and (B) late post-interval activity at fronto-central electrodes. (A) Mean amplitude at parietal-occipital electrodes (P8, P6, P4, P3, P5, P7, PO8, PO4, POz, PO3, PO7, O1, Oz, O2) in the 200ms to 300ms after S2 offset window, by proportional time and task. (B) Mean amplitude at fronto-central electrodes (F1, Fz, F2, FC1, FC2, C2, Cz, C1, CP1, CPz, CP2) in the 300ms to 500ms after S2 offset window. Faint-colored circles depict individual values, while sharp-colored circles represent the mean between subjects. Bars represent the standard error of the mean.

To illustrate the effects on these two ERPs, we calculated the mean amplitude of the early activity at parietal-occipital electrodes and the late activity at fronto-central electrodes (Fig 5). For the early parietal-occipital activity, ERP amplitudes seem to follow a direct monotonic relation to proportional time. On the other hand, for the late fronto-central activity, we found an inverse relationship between amplitude and proportional time in conditions in which time is relevant or possibly relevant. In these conditions, the larger the amplitude of the late ERP, the shorter is S2 relative to S1. In general, these results suggest that the modulation of this late fronto-central activity was strongly modulated by whether time was relevant or not to the current task.

#### The relation between post-interval activity, time, and behavior is task-dependent

To evaluate whether there is a relation between the post-interval activity and the response given by participants, we performed a binomial regression in which the binary answer (shorter or longer) was used as the response variable and the proportional time (5 values: 0.6, 0.8, 1.2, 1.4 and 1.8) and EEG activity residuals were explanatory variables. We performed this regression for all conditions for the two different sets of electrodes and the two windows after S2 offset (the same windows and electrodes explored at EEG activity topographies in Fig 4B). We calculated EEG activity residuals by subtracting the mean EEG amplitude for that specific proportion of S2 and condition in each electrode and time point. The residuals of EEG activity were used to minimize the correlation between proportional time and EEG activity. This allowed us to investigate whether trial-by-trial EEG fluctuations covaried with behavior.

As expected, proportional time was a significant predictor of behavior for all time-relevant conditions (Time Pure condition: first windows: t(20) = 11.63, p < .0001, d = 2.54, BF_10_ > 100 and second windows: t(20) = 11.49, p < .0001, d = 2.51, BF_10_ > 100; Time Mixed condition: first windows: t(20) = 12.64, p < .0001, d = 2.76, BF_10_ > 100 and second windows: t(20) = 12.56, p < .0001, d = 2.74, BF_10_ > 100), but not for color relevant conditions (Color Pure condition: first windows: t(20) = -0.46, p = 0.65, d = -0.10, BF_10_ = 0.25; and second windows: t(20) = -0.48, p = 0.63, d = -0.11, BF_10_ = 0.25; Color Mixed condition: first windows: t(20) = 0.88, p = 0.39, d = 0.19, BF_10_ = 0.32 and second windows: t(20) = 0.86, p = 0.40, d = 0.19, BF_10_ = 0.32).

Critically, early EEG post-interval activity weakly modulated behavior in the Time Mixed Condition (t(20) = 2.53, p = 0.02, d = 0.55, BF_10_ = 2.87), but not on other conditions (Color Mixed (t(20) = 1.55, p = 0.14, d = 0.34, BF_10_ = 0.63, Time Pure (t(20) = 1.18, p = 0.25, d = 0.26, BF_10_ = 0.42, Color Pure (t(20) = 0.41, p = 0.69, d = 0.09, BF_10_ = 0.24). On the other hand, late EEG post-interval activity was associated with temporal judgements for both Time Pure (t(20) = -2.97, p = 0.007, d = -0.65, BF_10_ = 6.35) and Time Mixed (t(20) = -2.34, p = 0.03, d = -0.51, BF_10_ = 2.04) but not for Color Mixed (t(20) = -1.67, p = 0.11, d = 0.36, BF_10_ = 0.75) nor Color Pure (t(20) = 0.46, p = 0.65, d = 0.10, BF_10_ = 0.25). In these results, the observed negative coefficients indicate that when EEG activity was higher than average for a given relative duration, participants had a higher probability of responding shorter.

## Discussion

In the present study, we investigated the neural correlates of temporal discrimination. To dissociate duration-related EEG from other task-related decisions, we used multivariate analyses to compare activity across two tasks: one in which participants had to compare the color of two stimuli and one they had to compare their durations. Across different blocks, participants did or did not have prior knowledge about what feature would have to be compared. As expected, our behavioral results showed a better performance when participants knew the feature to be compared.

Our task consisted of encoding duration/color information of a first stimulus and a decision about these features in a second stimulus. Using an MVPA approach, we investigated patterns of EEG activity, without needing to select moments and groups of sensors *a priori*, and compared whether and how EEG activity differed due to task-goals in these two phases. In general, we observed that: (1) During the encoding phase, EEG activity did not differ strongly across tasks; (2) During the decision phase, there were substantial differences in EEG activity across tasks and how task-relevant features modulated this activity.

Our task design allowed a temporal separation of when information about each feature was accessible to participants during the decision phase. While color information was available at the onset of S2, information about time was fully present only at its offset. This separation was reflected in the EEG signal, in which making decisions about color or time evoked activity at the onset and offset of S2, respectively. When making color decisions, there was a clear p300 at S2 onset, agreeing with proposals of this potential reflecting decision-making processes [28,29]. Although the p300 was not strongly modulated by how different S2 was relative to S1, this could be because the color task was slightly easier for participants. On the other hand, time-related information modulated activity more strongly in fronto-central sensors, at the offset of S2. Critically, this offset activity modulation by duration was most reliable when the temporal information was task-relevant and weaker when it was irrelevant or only possibly relevant. This result suggests that this late activity is not passively representing a possible surprise due to an interval ending, given that its presence seems to be directly related to this information being relevant to the given task.

Our findings partially agree with Kulashekhar et al. study [2] that did not find differences in the time-locked signal during the encoding period in MEG recordings. However, contrary to our findings, the authors did not find differences in the time-locked signal during the decision period. There were substantial differences between our tasks that might explain the contrast between these findings. In their work, the color task consisted of a varying hue that changed from a bluish to a reddish-purple during the trial. This was done as an attempt to make the color and the temporal task more comparable, given that participants would have to integrate information across the whole trial to make their color decision in a way similar to the temporal task [1,2]. Even with this control, it was possible that participants could still accumulate enough color information during S2, although in different moments across different trials [2], as suggested by the difference in reaction times between tasks. Another crucial difference is that temporal information could be used in the color discrimination task for participants to estimate the mean color. When evaluating the predominant color, participants can calculate for how long each hue was presented. On our task, the color discrimination task is not time-dependent, and thus the moment in which task-related information was made complete was clearer: at S2 onset for color and S2 offset for time. This allowed a more direct comparison and showed that decisions about time and color evoked different activity patterns.

An increasing number of studies have suggested that EEG markers at the end of the interval are correlated with temporal processing, such as the early post-interval N1P2 component [6] and the late positive component of timing [7–9]. In a recent study, Kruijne and colleagues [28] found a similar EEG pattern of a late positive signal amplitude (called P3 in their paper) and durations of the comparison stimuli. However, in their findings, this activity was not associated with behavior. On the other hand, the authors found that an early component (a fronto-central P2) was associated with behavior, with higher amplitudes related to more frequent long responses. Our results showed modulation of post intervals signals by time in EEG activity that resembled a parieto-occipital p200 and a later fronto-central similar to the LPC. However, only the LPC seemed to be more strongly correlated with time and behavior. The p200, on the other hand, seemed to be present irrespective of the task and was not associated with behavior.

Our results corroborate previous findings that the LPC might be related to decisional stages on other temporal tasks, such as temporal bisection [7,8,10], temporal generalization [7,11], and temporal discrimination [9,3,12–14]. However, it is important to stress that different studies have used LPC to refer to EEG activities diverse in time, topography, and task-related modulations. While some authors have measured the LPC to the response [7,8,10], others have measured it relative to the offset of the interval [3,9,12–14]. Additionally, different authors have identified the LPC at prefrontal electrodes [3,9,11], and centro-parietal electrodes [7,10]. In our results, the LPC that was more strongly modulated by temporal information had a fronto-central distribution and was inversely correlated with how much shorter the comparison interval was relative to the reference, in agreement with previous studies [7,8,10,28]. It is still unclear whether these different patterns are related to similar processing or capturing different temporal processing stages. Moreover, how the LPC is related to other decision-related components such as the p300 and the centro-parietal positivity (CPP) [29,30], and, more generally, whether a unique mechanism is used for different types of information integration or if a specific mechanism integrates time is still an open question that future studies can address more directly.

Recent proposals have approximated temporal processing with drift-diffusion models of decision-making [31–33]. When adapted to temporal discrimination tasks, these models posit that evidence accumulates towards one of two thresholds at the offset of the interval to be judged. Our findings are consistent with this proposal, with the pattern of accumulation possibly reflected on the LPC. Notably, the LPC in our results seemed to have a higher amplitude for shorter intervals than the reference, but not for intervals longer than the reference, similarly to other EEG results [7,8,10,28]. These findings suggest that the second stage occurs only for shorter than reference intervals, as indicated by previous behavioral results [34]. This proposal is contrary to the original model, in which a decision should always occur during the second stage [31–33]. However, our current study was not designed for behavioral modeling, and this hypothesis should be addressed in future studies. Finally, if a decision is made during the first stage for longer than reference trials, it might be possible to observe this decision during the stimulus itself. Future studies can focus on trying to identify the correlates of this decision.

In conclusion, our results suggest that task goals strongly modulate temporal information encoding in EEG activity. Future studies, using similar approaches, should investigate whether and how different temporal tasks modulate this activity pattern and whether it is present only in decisions about time or in other forms of decisions that evolve monotonically in time.

## Supporting information

S1 Fig Event-related potentials for the different task conditions at S1 onset for central electrodes. Grand average for central channels (FC1, FC2, C2, Cz, C1) for trials longer than 1300 ms at S1. Shaded areas depict the standard error of the mean.
(PDF)

S2 Fig Topographies by task at S1 onset. Mean topographies of the EEG signal in 130 ms windows during S1 onset for trials longer than 1300ms. Each row represents a different task condition.
(PDF)

S3 Fig Topographies by task at S1 offset. Mean topographies of the EEG signal in 66 ms windows from S1 offset to 400 ms. Each row represents a different task condition.
(PDF)

S4 Fig Time and color information at exposure. (Left) Color information at S1 onset. Coefficient estimates from RSA for color information at S1 onset by condition. (Right) Time information at S1 offset. Coefficient estimates from RSA for time information at S1 onset by condition. Lighter colors depict time bins of shorter intervals. Shaded areas depict the standard error of the mean.
(PDF)

S5 Fig Topographies by task at S2 onset. Mean topographies of the EEG signal in 130 ms windows during S2 onset for trials longer than 1300ms. Each row represents a different task condition.
(PDF)

S6 Fig Event-related potentials for the different task conditions at S2 onset for central electrodes. Grand average for central channels (FC1, FC2, C2, Cz, C1) for trials longer than 1300 ms at S2. Shaded areas depict the standard error of the mean.
(PDF)

S7 Fig Topographies by task at S2 offset. Mean topographies of the EEG signal in 70 ms windows from S1 offset to 500 ms. Each row represents a different task condition.
(PDF)

S8 Fig Event-related potentials for proportional color during S2 onset. Event-related potentials by proportional color from S2 to S1 at central-parietal electrodes for different conditions (channels: CP5, CP3, CP1, CPz, CP2, CP4, CP6, TP8, P8, P6, P4, P2, Pz, P1, P3, P5, P7). Shaded areas depict the standard error of the mean.
(PDF)

## References

[1] Coull JT, Vidal F, Nazarian B, Macar F. Functional anatomy of the attentional modulation of time estimation. Science 2004, 303(5663), 1506–1508. doi: 10.1126/science.1091573 15001776

[2] Kulashekhar S, Pekkola J, Palva JM, Palva S. The role of cortical beta oscillations in time estimation. Human brain mapping 2016, 37(9), 3262–3281. doi: 10.1002/hbm.23239 27168123 PMC6867325

[3] Gontier E, Le Dantec C, Paul I, Bernard C, Lalonde R, Rebai M. A prefrontal ERP involved in decision making during visual duration and size discrimination tasks. International Journal of Neuroscience 2008, 118(1), 149–162. doi: 10.1080/00207450601046798 18041612

[4] Coull JT, Charras P, Donadieu M, Droit-Volet S, Vidal F. SMA selectively codes the active accumulation of temporal, not spatial, magnitude. Journal of Cognitive Neuroscience 2015, 27(11), 2281–2298. doi: 10.1162/jocn_a_00854 26226079

[5] Schlichting N, de Jong R, van Rijn H. Performance-informed EEG analysis reveals mixed evidence for EEG signatures unique to the processing of time. Psychological research 2020, 84(2), 352–369. doi: 10.1007/s00426-018-1039-y 29926169 PMC7039843

[6] Kononowicz TW, van Rijn H. Decoupling interval timing and climbing neural activity: a dissociation between CNV and N1P2 amplitudes. Journal of Neuroscience 2014, 34(8), 2931–2939. doi: 10.1523/JNEUROSCI.2523-13.2014 24553934 PMC6608524

[7] Bannier D, Wearden J, Le Dantec C, Rebaï M. Differences in the temporal processing between identification and categorization of durations: a behavioral and ERP study. Behavioural brain research 2019, 356, 197–203. doi: 10.1016/j.bbr.2018.08.027 30189287

[8] Wiener M, Thompson JC. Repetition enhancement and memory effects for duration. Neuroimage 2015, 113, 268–278. doi: 10.1016/j.neuroimage.2015.03.054 25818689

[9] Paul I, Le Dantec C, Bernard C, Lalonde R, Rebaï M. Event-related potentials in the frontal lobe during performance of a visual duration discrimination task. Journal of clinical neurophysiology 2013, 20(5), 351–360. doi: 10.1097/00004691-200309000-00007 14701996

[10] Lindbergh CA, Kieffaber PD. The neural correlates of temporal judgments in the duration bisection task. Neuropsychologia 2013, 51(2), 191–196. doi: 10.1016/j.neuropsychologia.2012.09.001 22975193

[11] Paul I, Wearden J, Bannier D, Gontier E, Le Dantec C, Rebaï M. Making decisions about time: Event-related potentials and judgements about the equality of durations. Biological Psychology 2011, 88(1), 94–103. doi: 10.1016/j.biopsycho.2011.06.013 21756972

[12] Gontier E, Le Dantec C, Leleu A, Paul I, Charvin H, Bernard C, et al. Frontal and parietal ERPs associated with duration discrimination with or without task interference. Brain research 2007, 1170, 79–89. doi: 10.1016/j.brainres.2007.07.022 17706619

[13] Gontier E, Paul I, Le Dantec C, Pouthas V, Jean-Marie G, Bernard C, et al. ERPs in anterior and posterior regions associated with duration and size discriminations. Neuropsychology 2009, 23(5), 668. doi: 10.1037/a0015757 19702420

[14] Tarantino V, Ehlis AC, Baehne C, Boreatti-Huemmer A, Jacob C, Bisiacchi P, et al. The time course of temporal discrimination: an ERP study. Clinical Neurophysiology 2010, 121(1), 43–52. doi: 10.1016/j.clinph.2009.09.014 19914865

[15] Walther A, Nili H, Ejaz N, Alink A, Kriegeskorte N, Diedrichsen J. Reliability of dissimilarity measures for multi-voxel pattern analysis. Neuroimage 2016, 137, 188–200. doi: 10.1016/j.neuroimage.2015.12.012 26707889

[16] Kriegeskorte N, Mur M, Bandettini PA. Representational similarity analysis-connecting the branches of systems neuroscience. Frontiers in systems neuroscience 2008, 2, 4. doi: 10.3389/neuro.06.004.2008 19104670 PMC2605405

[17] Brainard DH. The psychophysics toolbox. Spatial vision 1997, 10(4), 433–436. 10.1163/156856897X00357. 9176952

[18] de Gardelle V, Summerfield C. Robust averaging during perceptual judgment. Proceedings of the National Academy of Sciences 2011, 108(32), 13341–13346. doi: 10.1073/pnas.1104517108 21788517 PMC3156162

[19] Oostenveld R, Fries P, Maris E, Schoffelen JM. FieldTrip: open source software for advanced analysis of MEG, EEG, and invasive electrophysiological data. Computational intelligence and neuroscience, 2011. 10.1155/2011/156869. PMC3021840 21253357

[20] Chaumon M, Bishop DV, Busch NA. A practical guide to the selection of independent components of the electroencephalogram for artifact correction. Journal of neuroscience methods 2015, 250, 47–63. doi: 10.1016/j.jneumeth.2015.02.025 25791012

[21] Wichmann FA, Hill NJ. The psychometric function: I. Fitting, sampling, and goodness of fit. Perception & psychophysics 2001, 63, 1293–313. 10.3758/BF03194544. 11800458

[22] Prins N, Kingdom FAA. Applying the Model-Comparison Approach to Test Specific Research Hypotheses in Psychophysical Research Using the Palamedes Toolbox. Frontiers in Psychology 2018, 9:1250. doi: 10.3389/fpsyg.2018.01250 30083122 PMC6064978

[23] JASP Team (2020). JASP (Version 0.12.2) [Computer software].

[24] Wolff MJ, Ding J, Myers NE, Stokes MG. Revealing hidden states in visual working memory using electroencephalography. Frontiers in Systems Neuroscience 2015, 9, 123. doi: 10.3389/fnsys.2015.00123 26388748 PMC4558475

[25] Ledoit O, Wolf M. Honey, I shrunk the sample covariance matrix. The Journal of Portfolio Management 2004, 30(4), 110–119. 10.3905/jpm.2004.110.

[26] Groppe DM, Urbach TP, Kutas M. Mass univariate analysis of event‐related brain potentials/fields I: A critical tutorial review. Psychophysiology 2011, 48(12), 1711–1725. doi: 10.1111/j.1469-8986.2011.01273.x 21895683 PMC4060794

[27] Tjur T. Coefficients of determination in logistic regression models—a new proposal: The coefficient of discrimination. The American Statistician 2009, 63(4):366–372. 10.1198/tast.2009.08210.

[28] Kruijne W, Olivers CN, van Rijn H. Neural Repetition Suppression Modulates Time Perception: Evidence From Electrophysiology and Pupillometry. J Cogn Neurosci 2021; 33 (7): 1230–1252. doi: 10.1162/jocn_a_01705 34496395

[29] O’connell RG, Dockree PM, Kelly SP. A supramodal accumulation-to-bound signal that determines perceptual decisions in humans. Nature Neuroscience 2012, 15(12), 1729. doi: 10.1038/nn.3248 23103963

[30] Twomey DM, Murphy PR, Kelly SP, O’Connell, RG. The classic P300 encodes a build‐to‐threshold decision variable. European journal of neuroscience 2015, 42(1), 1636–1643. 10.1111/ejn.12936. 25925534

[31] Balcı F, Simen P. Decision processes in temporal discrimination. Acta Psychologica 2014, 149, 157–168. doi: 10.1016/j.actpsy.2014.03.005 24726447

[32] Balcı F, Simen P. A decision model of timing. Current Opinion in Behavioral. Sciences 2016, 8:94–101. 10.1016/j.cobeha.2016.02.002.

[33] Simen P, Balci F, de Souza L, Cohen JD, Holmes P. A model of interval timing by neural integration. Journal of Neuroscience 2011, 31(25), 9238–9253. doi: 10.1523/JNEUROSCI.3121-10.2011 21697374 PMC3142662

[34] Wiener M, Parikh A, Krakow A, Coslett HB. An intrinsic role of beta oscillations in memory for time estimation. Scientific reports 2018; 8(1), 1–17. doi: 10.1038/s41598-017-17765-5 29789611 PMC5964239

