# Supplementary material for: Post-interval EEG activity is related to task-goals in temporal discrimination
Source: PLoS One. 2021 Sep 27;16(9):e0257378. doi: 10.1371/journal.pone.0257378 (PMC8476012; doi:10.1371/journal.pone.0257378)
Supplement: S1 Fig — Grand average for central channels (FC1, FC2, C2, Cz, C1) for trials longer than 1300 ms at S1. Shaded areas depict the standard error of the mean. (PDF) [file pone.0257378.s001.pdf]

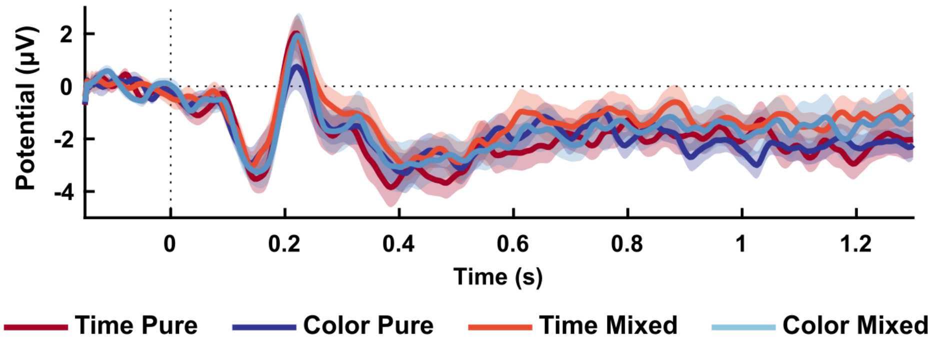

Fig S1. **Event-related potentials for the different task conditions at S1 onset for central electrodes.** Grand average for central channels (FC1, FC2, C2, Cz, C1) for trials longer than 1300 ms at S1. Shaded areas depict the standard error of the mean.
