# Supplementary material for: Post-interval EEG activity is related to task-goals in temporal discrimination
Source: PLoS One. 2021 Sep 27;16(9):e0257378. doi: 10.1371/journal.pone.0257378 (PMC8476012; doi:10.1371/journal.pone.0257378)
Supplement: S2 Fig — Mean topographies of the EEG signal in 130 ms windows during S1 onset for trials longer than 1300ms. Each row represents a different task condition. (PDF) [file pone.0257378.s002.pdf]

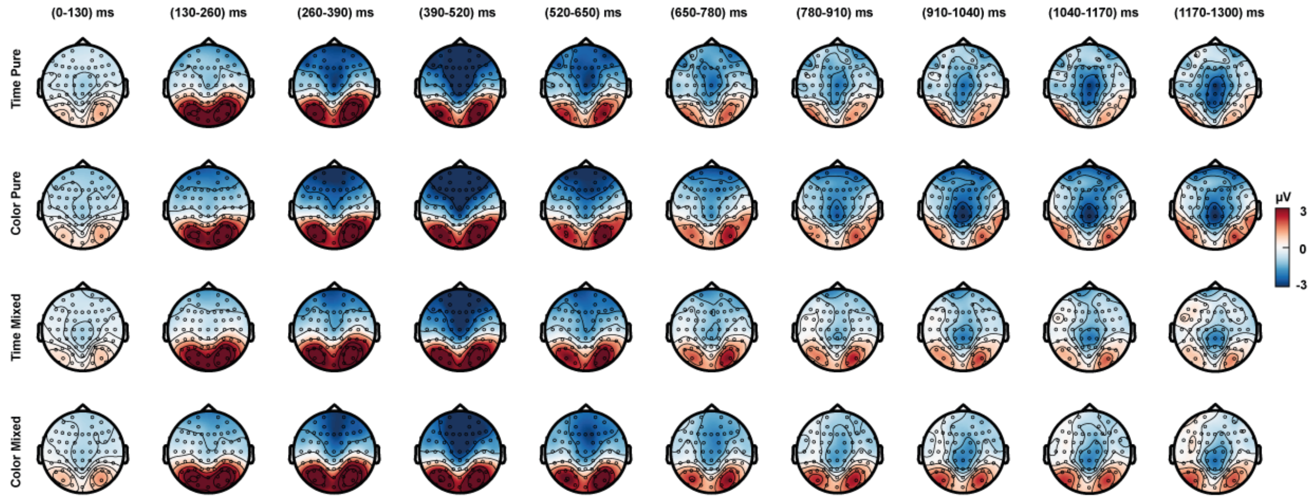

Fig S2. Topographies by task at S1 Onset. Mean topographies of the EEG signal in 130 ms windows during S1 onset for trials longer than 1300ms. Each row represents a different task condition.
