# Supplementary material for: Post-interval EEG activity is related to task-goals in temporal discrimination
Source: PLoS One. 2021 Sep 27;16(9):e0257378. doi: 10.1371/journal.pone.0257378 (PMC8476012; doi:10.1371/journal.pone.0257378)
Supplement: S4 Fig — (Left) Color information at S1 onset. Coefficient estimates from RSA for color information at S1 onset by condition. (Right) Time information at S1 offset. Coefficient estimates from RSA for time information at S1 onset by condition. Lighter colors depict time bins of shorter intervals. Shaded areas depict the standard error of the mean. (PDF) [file pone.0257378.s004.pdf]

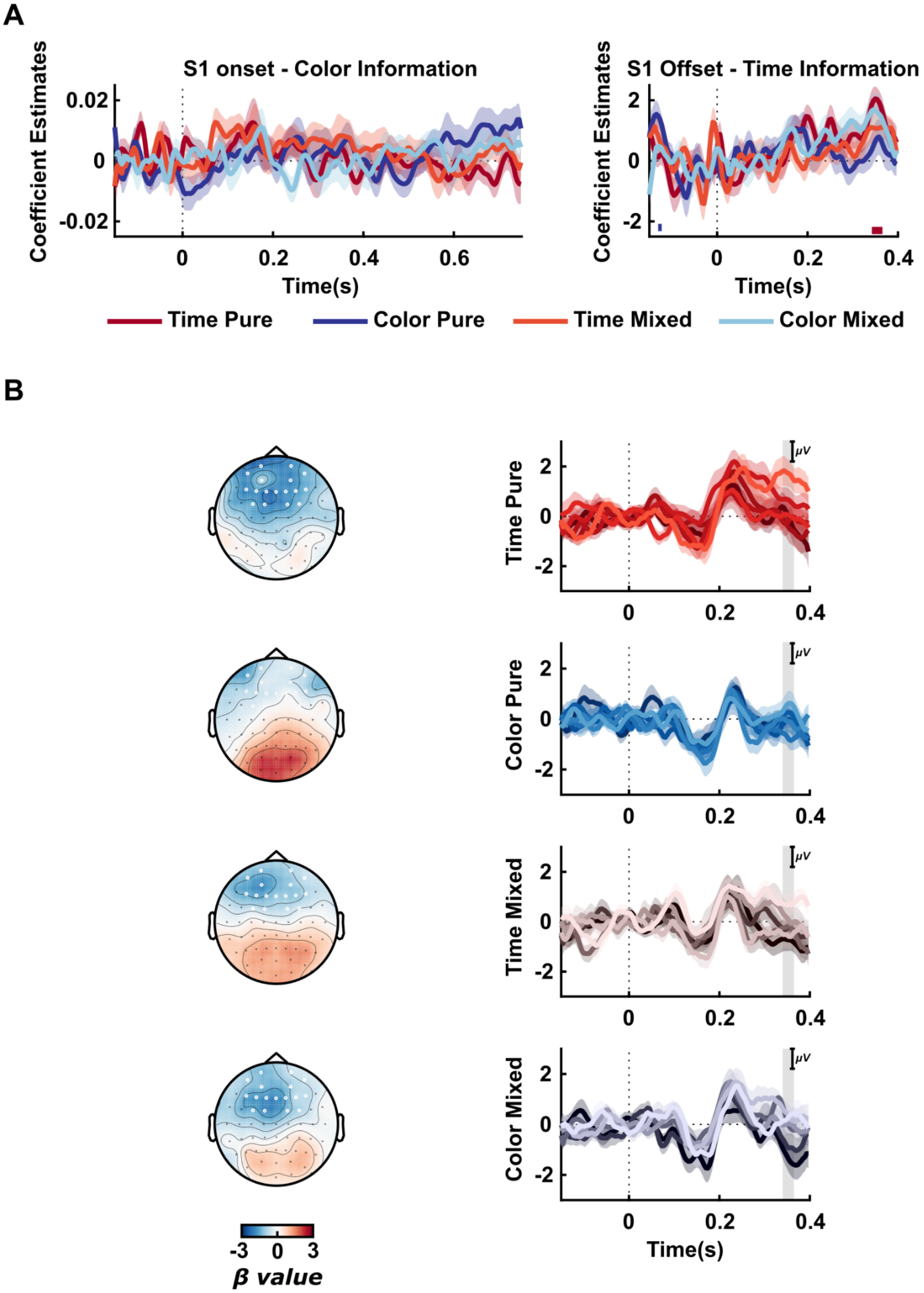

Fig S4. **Time and Color Information at exposure.** (Left) Color information at S1 onset. Coefficient estimates from RSA for color information at S1 onset by condition. (Right) Time information at S1 offset. Coefficient estimates from RSA for time information at S1 onset by condition. Lighter colors depict time bins of shorter intervals. Shaded areas depict the standard error of the mean.
