# Supplementary material for: Post-interval EEG activity is related to task-goals in temporal discrimination
Source: PLoS One. 2021 Sep 27;16(9):e0257378. doi: 10.1371/journal.pone.0257378 (PMC8476012; doi:10.1371/journal.pone.0257378)
Supplement: S7 Fig — Mean topographies of the EEG signal in 70 ms windows from S1 offset to 500 ms. Each row represents a different task condition. (PDF) [file pone.0257378.s007.pdf]

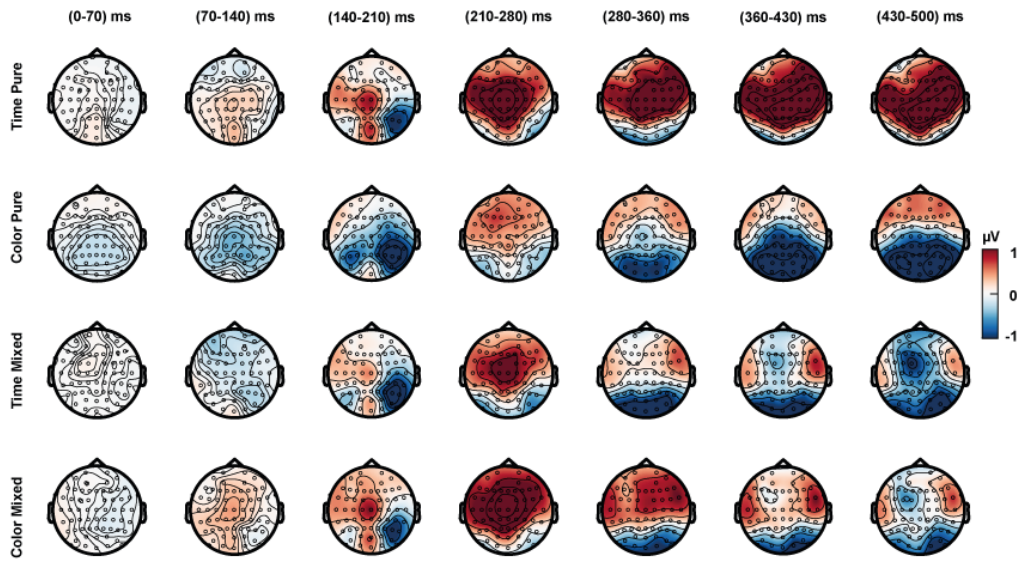

Fig S7. **Topographies by Task at S2 Offset.** Mean topographies of the EEG signal in 70 ms windows from S1 offset to 500 ms. Each row represents a different task condition.
