# Supplementary material for: Post-interval EEG activity is related to task-goals in temporal discrimination
Source: PLoS One. 2021 Sep 27;16(9):e0257378. doi: 10.1371/journal.pone.0257378 (PMC8476012; doi:10.1371/journal.pone.0257378)
Supplement: S8 Fig — Event-related potentials by proportional color from S2 to S1 at central-parietal electrodes for different conditions (channels: CP5, CP3, CP1, CPz, CP2, CP4, CP6, TP8, P8, P6, P4, P2, Pz, P1, P3, P5, P7). Shaded areas depict the standard error of the mean. (PDF) [file pone.0257378.s008.pdf]

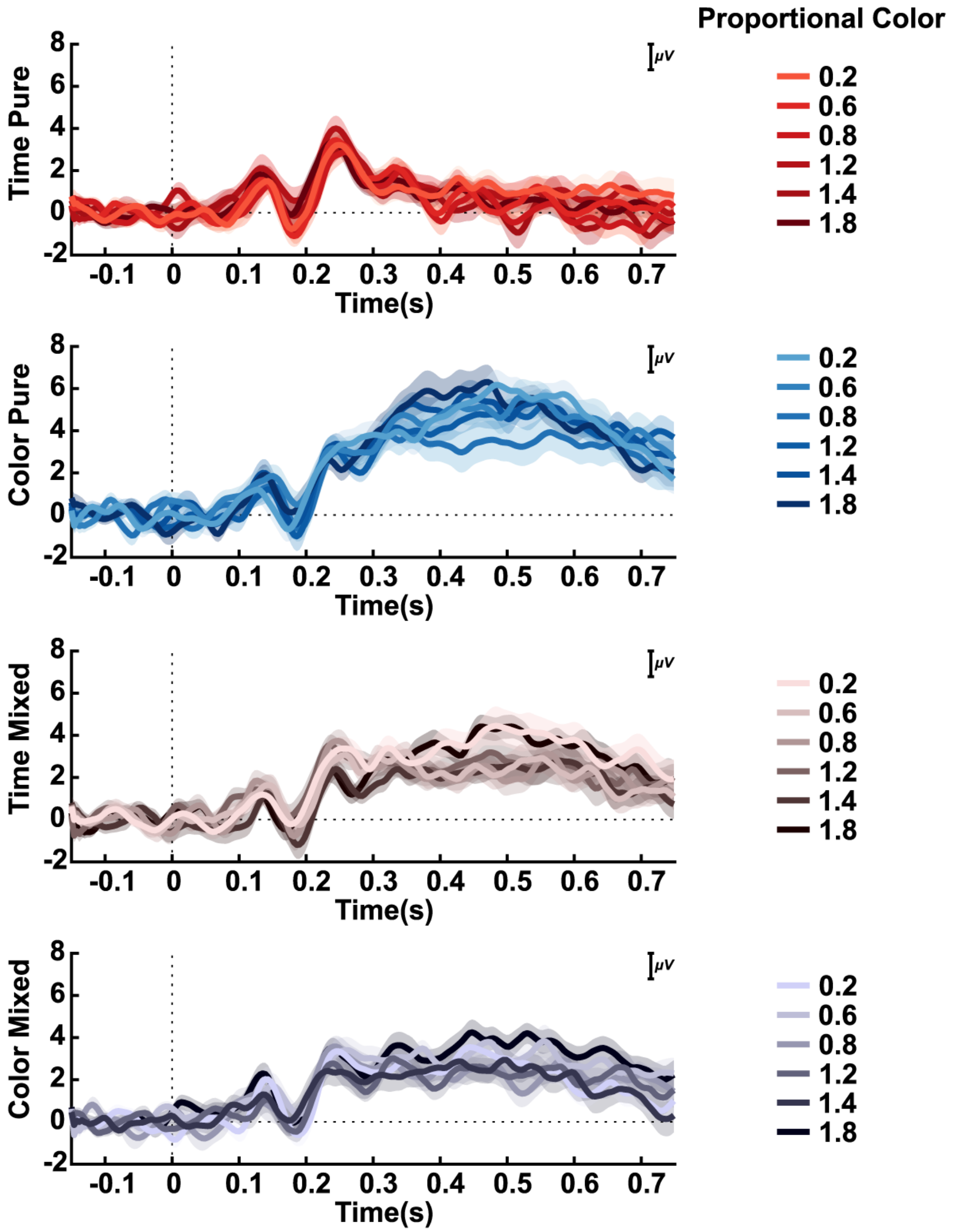

Fig S8. **Event-related potentials for proportional color during S2 onset.** Event-related potentials by proportional color from S2 to S1 at central-parietal electrodes for different conditions (channels: CP5, CP3, CP1, CPz, CP2, CP4, CP6, TP8, P8, P6, P4, P2, Pz, P1, P3, P5, P7). Shaded areas depict the standard error of the mean.
